# Primary catastrophic antiphospholipid syndrome in children with midbrain infarction: a case report

**DOI:** 10.3389/fped.2024.1370843

**Published:** 2024-04-08

**Authors:** Qinghua Dong, Jianyun Yin, Hang Su, Qian Ni

**Affiliations:** ^1^ The Second Clinical Medical College of Lanzhou University, Lanzhou University, Lanzhou, China; ^2^ Pediatric Respiratory Medicine, Lanzhou University Second Hospital, Lanzhou, China

**Keywords:** catastrophic antiphospholipid syndrome, brain stem infarction, pediatric, thrombosis, chorea

## Abstract

**Background:**

Catastrophic antiphospholipid syndrome (CAPS) is a multi-system autoimmune disease characterized by extensive thrombosis. Pediatric CAPS is extremely rare and associated with a high mortality rate, especially when midbrain infarction is involved. Hence, early diagnosis and prompt initiation of appropriate treatment for CAPS complicated by midbrain infarction are of utmost importance in achieving favorable outcomes.

**Case presentation:**

In this report, we present the case of a 14-year-old girl who presented with neurological symptoms and digestive system infection and was initially diagnosed with an “intracranial infection”. After a series of rigorous diagnostic procedures, the patient was ultimately diagnosed with primary CAPS and was immediately transferred to the intensive care unit where she was treated with anticoagulation, glucocorticoids, intravenous immunoglobulin (IVIG) therapy, and multiple plasma infusions. Twenty-seven days after admission, the patient's condition improved with standardized treatment, and she was discharged and followed up regularly.

**Conclusion:**

This case report provides a description of the clinical features observed in a pediatric patient with CAPS and concurrent midbrain infarction, highlighting the crucial role of early diagnosis and timely treatment in influencing patient prognosis.

## Background

Catastrophic antiphospholipid syndrome (CAPS) is a severe form of antiphospholipid syndrome (APS) characterized by the occurrence of multiple thrombotic events within a short period of time, accompanied by elevated titers of antiphospholipid antibodies (aPL)—including persistently positive lupus anticoagulant, anti-β2-glycoprotein, and anti-cardiolipin antibodies—often leading to multi-organ failure and life-threatening conditions. While CAPS is well documented in adults, it is extremely rare in children; however, it is known to be associated with a high mortality rate (33%–50%) (1). Previous studies have shown minimal differences in clinical and laboratory characteristics, treatment, and outcomes between pediatric and adult patients with CAPS. The primary difference is that pediatric populations have a higher proportion of infections as triggering factors than adults (2). It is worth noting that although the proportion of primary diseases in pediatric APS is low, the opposite is often observed in CAPS (38%–50% vs. 70%, respectively), with nearly 90% of pediatric APS cases presenting as CAPS at the time of disease onset (2, 3).

The general diagnostic criteria for CAPS is the following (1) the presence of persistent aPL, such as anti-cardiolipin antibodies, anti-*β*2-glycoprotein I antibodies (*β*2GPIA), and/or lupus anticoagulant (LA), in titers higher than 40 UI/L that have been detected for at least 12 weeks, and (2) evidence of the involvement of at least three organs, organ systems, and/or tissues, with these injuries occurring within 1 week. After ruling out other diagnoses, the diagnosis of CAPS can then be made. In the pediatric population, apart from being vigilant for thrombus formation in various organs or tissues, it is important to consider the differential diagnosis of non-criteria manifestations, as many pediatric cases of CAPS present as APS, which often has non-thrombotic manifestations. This means that the observation of thrombus formation in the early disease stages may not be sufficient to make a diagnosis. While central nervous system involvement frequently manifests as chorea, epilepsy, migraine, pseudotumor cerebri, mood disorder, cognitive disorder, and transverse myelitis, skin involvement frequently manifests as livedo reticularis, Raynaud phenomenon, skin ulcers, and purpura fulminans. Finally, blood system involvement frequently manifests as thrombocytopenia, autoimmune hemolytic anemia, Evans syndrome, leukopenia, or bleeding disorders (1, 4–6).

The treatment for pediatric CAPS is based off of adult guidelines and generally involves a multimodal therapy regimen comprising anticoagulants, intravenous corticosteroids, plasma replacement, and/or intravenous immunoglobulins. However, some researchers have suggested that only anticoagulation (AC) has a significant impact on the disease prognosis in these patients. Additionally, rituximab and eculizumab may be administered in refractory cases (7). Despite standardized treatment, the mortality rate associated with this disease remains high (up to 30%) (6).

In the pediatric intensive care unit (PICU) setting, the rarity and high mortality rate, combined with non-thrombotic manifestations in pediatric patients, can lead to misdiagnosis of the disease and missed detection of thrombosis in critical areas, which can be fatal for the patients. Through this case report, we aim to raise awareness among clinicians about the possibility of brainstem infarction in children with CAPS, especially when non-criteria symptoms resembling chorea are present.

## Case presentation

A 14-year-old girl presented to the pediatric emergency department with cognitive impairment and chorea-like movements due to a digestive system infection. According to her mother, the patient had experienced fever, abdominal pain, and diarrhea for 2 weeks prior to admission. After 5 days of antibiotic treatment at a local hospital, her symptoms improved but cognitive impairment and chorea-like movements had emerged leading to her admission to our hospital. Shortly after transfer, the patient's consciousness deteriorated and she went into a coma. Her GCS score was eight, with response to stimuli but without verbal response, and withdrawal response to painful stimuli. The patient had no history of autoimmune diseases, cerebrovascular events, or seizures.

**Figure 1:**
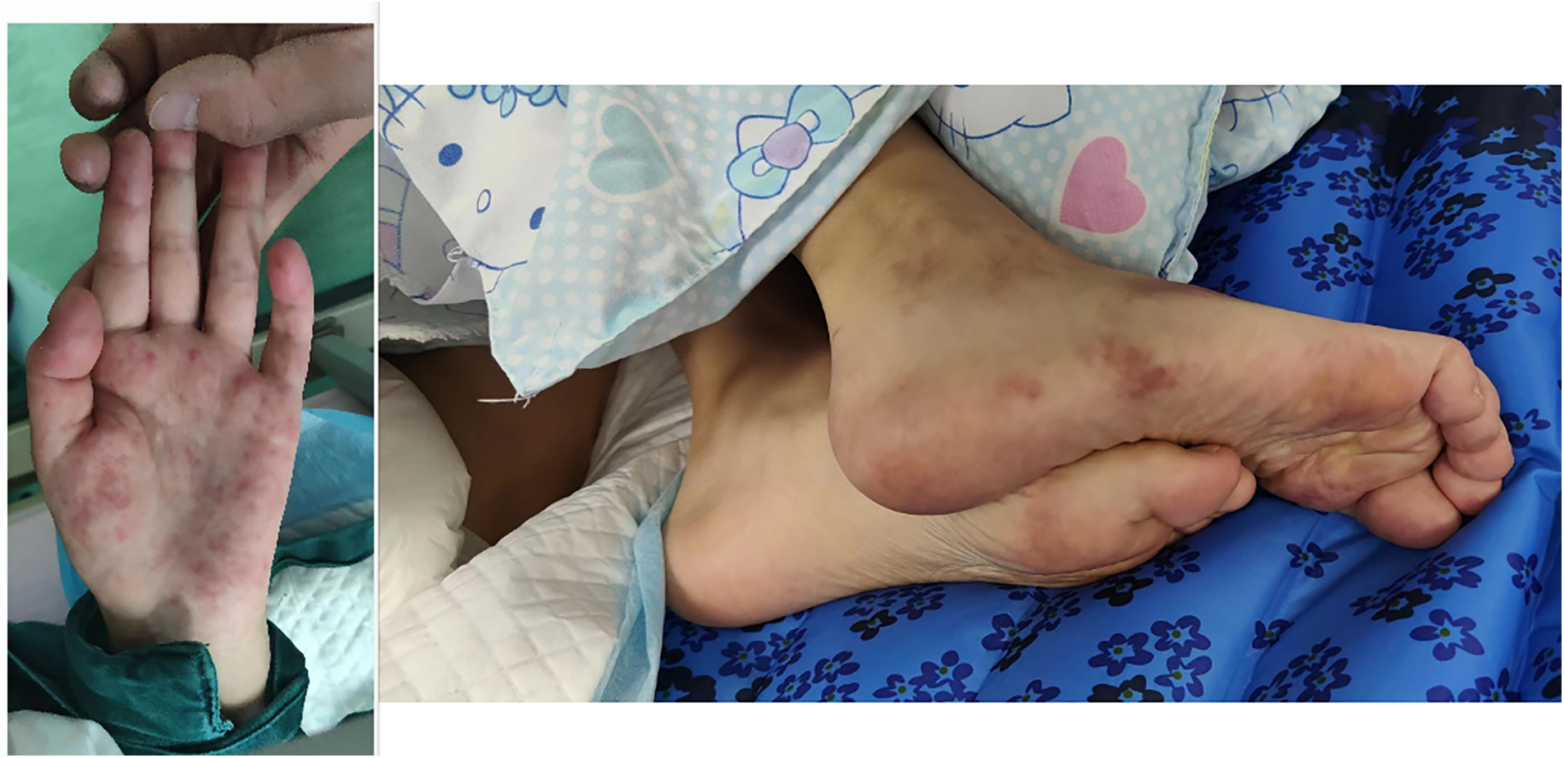
Microthrombus formation was also observed in the patient's palms and feet.

**Figure 2:**
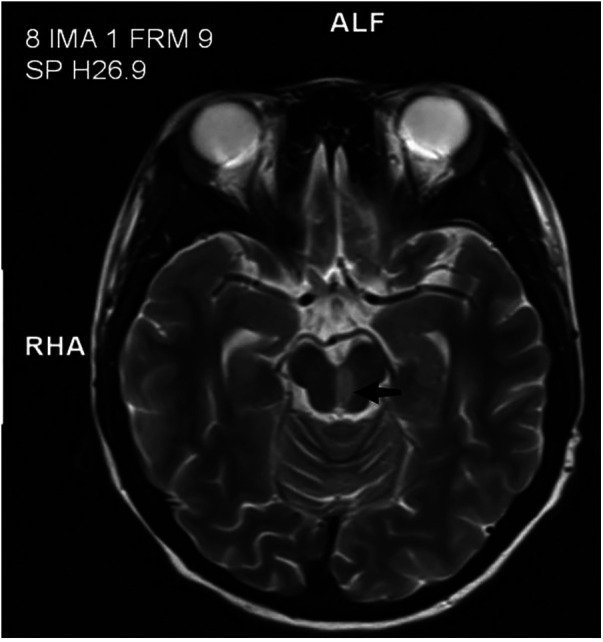
Left-sided infarct in the midbrain.

Physical examination revealed she had a low body weight (only 31 kg), cognitive impairment, and large and rapid involuntary movements in the right limbs motor aphasia was also present. The initial diagnosis was “intracranial infection”. After three days of prescribed IVIG therapy, glucocorticoid treatment and antibiotic use, the patient’s level of consciousness (LOC) improved. However, the patient still had choreiform movements and developed purpuric rashes on her hands and feet, that did not fade with added pressure (Figure 1). Head magnetic resonance imaging (MRI) revealed a left-sided infarct in the midbrain (Figure 2). Chest computed tomography (CT) revealed a bilateral pulmonary embolism in the basal regions of the lungs. Combined with the appearance of the rash on the palms and soles of the feet, we could not rule out multiple system vascular embolism. Therefore, a comprehensive multi-system evaluation was performed on the patient (Table 1). Doppler ultrasound examination did not reveal significant formation of cardiac, renal, or limb vascular thrombi, except for mild pyelectasis in the kidneys. Gastroscopy revealed erosive gastritis with gastroesophageal reflux and erosive esophagitis. Due to lack of patient's parents consent, genetic testing was not conducted. We also analyzed the cause of the patient's anemia, and a positive Coombs test indicated the presence of hemolytic anemia. Despite the patient's low mean corpuscular volume (MCV) suggesting microcytic anemia, we believe it is likely related to iron deficiency due to long-term malnutrition. Since no definitive evidence of thrombosis or vasculitis was found, we were unable to assess whether acute pancreatitis and acute gastritis were related to CAPS, after ruling out other autoimmune diseases and considering the background of widespread thrombosis, we speculate that they may be associated with microthrombosis in CAPS. Rheumatological tests included ANA antibodies, dsDNA was condycted to rule out secondary diseases, we confirmed the primary diagnosis of CAPS. The patient received 27 days of treatment in the PICU, with interdisciplinary discussions occurring among the medical team. In terms of treatment, the patient received standardized triple therapy with anticoagulation, glucocorticosteroids, and IVIG therapy. However, when considering plasma replacement, we considered other factors such as the patient’s low body weight, anemia, and disseminated intravascular coagulation (DIC). We adopted multiple plasma transfusions as an alternative treatment and observed that her aPL level had significantly decreased. As a result, the patient's cognitive impairment was significantly improved, her choreiform movements disappeared, and significant neurological sequelae were also not observed after treatment. In addition, during the observation period with regular follow-ups once a month for 6 months, the patient did not have any recurrence of the disease.

**Table 1:** The patient's laboratory data ranged from admission to 6 months after discharge.

Test	Timeline			Normal
	At the time of admission	At the time of discharge	6 months after discharge	
Hemoglobin	6.7	10.8	11.1	11.4–15.4 g/dl
Platelet	152	176	251	105–407*10^9 ^/L
D-dimer	6.38	0.25	/	<0.5 ug/ml
APTT	71.8	54.5	/	25.4–38.4 s
MCV	66.8	68.1	64.2	80–100 fl
ferritin	642	302	128	13–150 ng/ml
Creatinine	57	41.7	48.2	33.0–75.0 umol/L
Urea	6.7	3.6	3	2.5–6.5 mmol/L
Amylase	498	/	/	25–125 U/L
Lipase	>2,000	/	/	<190 U/L
ASO	174.6	/	/	<200 IU/ml
dsDNA	Negative	Negative	Negative	Negative
ANA	Negative	Negative	Negative	Negative
IgG aCL	112.1	26.5	103.5	<12R U/ml
IgA aCL	<12	<12	<12	<12R U/ml
IgM aCL	34.5	37.4	35.3	<12R U/ml
anti-*β*2-glycoprotein I	>200	>200	>200	<20R U/ml
Lupus anticoagulant ratio	2.37	1.93	2.08	<1.2

## Discussion

CAPS is an exceptionally rare yet morbid disease among the pediatric population. This condition is characterized by the combined impairment of multiple organ functions, distinguishing it from APS. However, owing to its rarity, there is a limited literature that explores CAPS in pediatric patients. Only one recent case report described ischemic stroke as a coincidental clinical presentation of pediatric CAPS, but specifically presented CAPS as a secondary manifestation in the setting of systemic lupus erythematosus (SLE) (8). Compared to that case, our case had a smaller infarction area and a better prognosis.

During the patient's emergency department visit, she was not immediately diagnosed with CAPS. We believe this was in part due to the unfamiliarity of the disease amongst the primary healthcare providers, as it is extremely rare in both the general population and the pediatric population. It is crucial for emergency and pediatric clinicians to be aware of this potential diagnosis, as it is may present in pediatric patients with neurological symptoms due to a digestive system infection. Failure to correctly diagnose CAPS can lead to worsening of the condition and potentially fatal consequences for the child.

An analysis of 45 children with catastrophic APS from the CAPS Registry revealed that cerebral involvement was present in 22 cases (47.8%) (2), including manifestations such as encephalopathy, cerebrovascular accidents, and seizures. In our patient, cerebral involvement was indicated by choreiform movement and the presence of a midbrain infarction. Although choreiform movement can be seen in cases without significant thrombotic events, midbrain infarction often leads to a series of manifestations such as extrapyramidal dysfunction, loss of consciousness, and incontinence. Therefore, we attribute the patient's choreiform symptoms to extrapyramidal dysfunction caused by midbrain infarction. Overall, we believe that central nervous system pathology cannot be separated from CAPS and that when corresponding neuropsychiatric symptoms and cerebral involvement are found, it is necessary to closely monitor patients due to the possibility of corresponding thrombotic events in the brain.

This case has provided valuable insight into the potentially close relationship between non-criteria manifestations and thrombus formation in pediatric CAPS, which is of utmost importance in guiding clinical diagnosis and treatment. Typically, in pediatric cases of APS, non-thrombotic clinical manifestations (such as thrombocytopenia) and neurological disorders (such as migraine, epilepsy, and chorea) may precede thrombotic manifestations (9). Even without clear evidence of thrombotic formation in the brain, choreiform movement has been identified as a characteristic manifestation of pediatric APS (10). However, our case presents opposite findings, as the presence of chronic midbrain infarction on MRI suggests thrombus formation prior to the occurrence of neurological manifestations and may likely be the underlying cause of these neurological conditions. Therefore, it is crucial to emphasize considering the possibility of midbrain infarction in children with CAPS who present with altered consciousness and chorea-like movements.

Triple therapy with anticoagulation, corticosteroids, and plasma replacement or IVIG administration has been proposed as an optimal treatment strategy for CAPS (11). Although plasma replacement is an established treatment option and a standard procedure in adult CAPS, we were unable to evaluate whether the pre-bypass process of priming the circuit would cause further harm to the patient owing to her low body weight and hemodynamic instability. Additionally, clinical evidence supporting the application of plasma replacement therapy in pediatric patients with CAPS is lacking. Therefore, after a detailed assessment of the patient's condition, we empirically administered multiple plasma infusions as an alternative to plasma replacement, considering that multiple plasma infusions reduce the amount of plasma used and clinical risks associated with plasma replacement. This approach may serve as a reference for the treatment of children with a low blood volume. However, this empirical approach still requires further clinical observation.

## Conclusion

While CAPS is exceedingly rare in the pediatric population, this case suggests that PICU physicians must be aware of its existence during the diagnostic and treatment processes, as misdiagnosis or delayed diagnosis poses significant risks to the patient's life. Furthermore, we advocate for more standardized diagnostic and treatment protocols for pediatric CAPS to facilitate earlier recognition and uniform management in clinical practice. Finally, we emphasize considering the possibility of midbrain infarction in children with CAPS who present with altered consciousness and chorea-like movements, rather than merely classifying them as atypical CAPS manifestations.

## Data Availability

The datasets presented in this study can be found in online repositories. The names of the repository/repositories and accession number(s) can be found in the article/Supplementary Material.

## References

[1] Go E O'Neil KM. The catastrophic antiphospholipid syndrome in children. Curr Opin Rheumatol. (2017) 29(5):516–22. 10.1097/BOR.0000000000000426 28632503

[2] Berman H Rodríguez-Pintó I Cervera R Gregory S de Meis E Rodrigues CE Pediatric catastrophic antiphospholipid syndrome: descriptive analysis of 45 patients from the “CAPS registry”. Autoimmun Rev. (2014) 13(2):157–62. 10.1016/j.autrev.2013.10.004 24145009

[3] Wincup C Ioannou Y. The differences between childhood and adult onset antiphospholipid syndrome. Front Pediatr. (2018) 6:362. 10.3389/fped.2018.00362 30542645 PMC6277799

[4] Barbhaiya M Zuily S Naden R Hendry A Manneville F Amigo MC 2023 ACR/EULAR antiphospholipid syndrome classification criteria. Ann Rheum Dis. (2023) 82(10):1258–70. 10.1136/ard-2023-224609 37640450

[5] Cervera R Rodriguez-Pinto I Espinosa G. The diagnosis and clinical management of the catastrophic antiphospholipid syndrome: a comprehensive review. J Autoimmun. (2018) 92:1–11. 10.1016/j.jaut.2018.05.007 29779928

[6] Islabão AG Trindade VC da Mota LMH Andrade DCO Silva CA. Managing antiphospholipid syndrome in children and adolescents: current and future prospects. Paediatr Drugs. (2022) 24(1):13–27. 10.1007/s40272-021-00484-w 34904182 PMC8667978

[7] Kazzaz NM McCune WJ Knight JS. Treatment of catastrophic antiphospholipid syndrome. Curr Opin Rheumatol. (2016) 28(3):218–27. 10.1097/BOR.0000000000000269 26927441 PMC4958413

[8] Senken B Whitehead A. Catastrophic antiphospholipid syndrome presenting as a stroke in an 11-year-old with lupus. Case Rep Pediatr. (2022) 2022:7890566. 10.1155/2022/7890566 35600982 PMC9122718

[9] Soybilgic A Avcin T. Pediatric APS: state of the art. Curr Rheumatol Rep. (2020) 22(3):9. 10.1007/s11926-020-0887-9 32124078

[10] Avcin T Cimaz R Silverman ED Cervera R Gattorno M Garay S Pediatric antiphospholipid syndrome: clinical and immunologic features of 121 patients in an international registry. Pediatrics. (2008) 122(5):e1100–7. 10.1542/peds.2008-1209 18955411

[11] Rodríguez-Pintó I Lozano M Cid J Espinosa G Cervera R. Plasma replacement in catastrophic antiphospholipid syndrome. Presse Med. (2019) 48(11 Pt 2):347–53. 10.1016/j.lpm.2019.10.003 31694791

